# Can we predict left ventricular dysfunction-induced weaning failure? Invasive and echocardiographic evaluation

**DOI:** 10.1186/cc9584

**Published:** 2011-03-11

**Authors:** A Abdelbary, W Ayoub, Y Nassar, K Hussein

**Affiliations:** 1 Faculty of Medicine, Cairo University, Cairo, Egypt

## Introduction

The aim was to study the relation of weaning failure to development of diastolic dysfunction using echocardiography and PA catheter.

## Methods

Thirty invasively mechanically ventilated patients fulfilling criteria of weaning from mechanical ventilation were shifted to SBT (using low PSV (8 cmH_2_O)) for 30 minutes. Two sets of variables were measured at the beginning and end of the SBT. Weaning failure was defined as: failed SBT, reintubation and/or ventilation or death within 48 hours following extubation. A Swan-Ganz catheter was used to obtain the right atrial (RAP), pulmonary artery (PAP), pulmonary artery occlusion (PAOP) pressures, and cardiac index (CI). Echocardiography: the LV internal diameter at end diastole (LVIDd) and end systole (LVIDs), ejection fraction (LVEF), E/A ratio, deceleration time (DT) (ms), isovolumetric relaxation time (IVRT), and E/E' ratio.

## Results

Mean age was 56.6 ± 15.9 years, 53% were males. The outcome of weaning was successful in 76.6% of patients. The patients were subdivided into two groups according to weaning outcome: Group I, 23 patients (successful weaning); Group II, seven patients (failed weaning). RAP, PAOP and SVO_2 _were similar at the start of SBT (6.3 ± 1.9 vs. 7.6 ± 2.3, *P *= 0.1; 12 ± 3.7 vs. 14.6 ± 3, *P *= 0.4; 72 ± 2.4 vs. 71 ± 3.1, *P *= 0.1) between Groups I and II yet significantly different at the end (6.2 ± 2.4 vs.10 ± 3.5, *P *= 0.01; 12.8 ± 3.5 vs.19 ± 5.4, *P *= 0.004; 73 ± 2.8 vs. 66.6 ± 7, *P *= 0.009), respectively. CI was similar between Groups I and II at both ends of the SBT, *P *= 0.5 and *P *= 0.9. Groups I and II had similar LVIDs and EF at the beginning of SBT (3 ± 0.7 vs. 3.3 ± 0.5, *P *= 0.2; 68 ± 8 vs. 62 ± 6, *P *= 0.08) yet different at the end (3 ± 0.6 vs. 3.5 ± 0.5, *P *= 0.048; 66 ± 8 vs. 58 ± 7, *P *= 0.03), respectively. There was no significant differences in E/A, IVRT, DT yet a significant difference in E/E' between Group I and Group II at both ends of the trial (1.04 ± 0.4 vs. 0.97 ± 0.3, *P *= 0.78; 1.02 ± 0.4 vs. 1.07 ± 0.4, *P *= 0.78; 94 ± 26 vs. 99.6 ± 18, *P *= 0.52; 97 ± 22 vs. 91 ± 24, *P *= 0.57; 194 ± 31 vs. 196 ± 30, *P *= 0.98; 197 ± 27 vs. 189 ± 33, *P *= 0.6; 8.9 ± 2 vs. 12.2 ± 4, *P *= 0.02; 9.4 ± 2.3 vs. 13 ± 5, *P *= 0.02), respectively.

## Conclusions

LV dysfunction may have an impact on weaning outcome. Invasive monitoring as well as echocardiography and tissue Doppler indices may be reliable in monitoring and detection of LV dysfunction, and subsequently may be possibly useful in improving weaning outcome. RAP may be a particularly reliable and easy method to monitor during the period of weaning.

